# ‘We’re not there yet!’: a qualitative study exploring the commissioning of adult Community Health Services in England to support the avoidance of hospital admissions

**DOI:** 10.1136/bmjopen-2024-098159

**Published:** 2025-05-31

**Authors:** Donna Bramwell, Mhorag Goff, Pauline Allen, Rachel Meacock, Kath Checkland

**Affiliations:** 1 Centre for Primary Care and Health Services Research, The University of Manchester Faculty of Biology Medicine and Health, Manchester, UK; 2 Health Services Research and Policy, London School of Hygiene & Tropical Medicine, London, UK

**Keywords:** Health Services, Health policy, Organisation of health services

## Abstract

**Abstract:**

**Objectives:**

The increased use of Community Health Services (CHS) is central to UK policy visions of moving more care out of hospital to reduce pressure across the healthcare system and, in particular, the demand on secondary care, hospital services. CHS are under-researched, and little is known about how they can best contribute towards this aim. The National Health Service (NHS) in England has recently undergone a significant reorganisation, with an increased emphasis on collaborative service delivery. In the aftermath of this reorganisation, the objective of this study was to explore how commissioners and providers of CHS think about the need for services and how decisions are made about the commissioning and allocation of resources in order to facilitate out-of-hospital care.

**Design:**

A qualitative, semi-structured interview study with participants from four case study sites in England. Semi-structured interviews were conducted virtually and transcripts analysed using a reflexive thematic approach.

**Setting:**

Adult CHS, which included two sites with CHS providers embedded in acute hospital Trusts, one standalone CHS Trust and a CHS provider collaborative. Sites were selected for both geographical (two sites in the north of England and two in the South) and organisational model diversity.

**Participants:**

40 participants were interviewed across all four case study sites (site A, n=10; site B, n=17; site C, n=10; and site D, n=3). To be included in the study, participants were required to have a management role in providing or commissioning adult CHS and/or their understanding of this at strategic level within the Integrated Care Systems.

**Results:**

Themes from current literature on commissioning (organisation, assessing needs, service design and development, contracting and funding, and performance management and support) were used to structure the data. Participants from all sites reported that the reorganisation of the NHS away from Clinical Commissioning Groups to Integrated Care Boards has resulted in confusion around the commissioning function, with a lack of clarity about current roles and responsibilities. All sites were undertaking some form of service review. However, participants highlighted the fact that current population health and CHS service data do not adequately support proactive planning of services to meet rising demand. CHS find it particularly difficult to evidence their contribution to hospital avoidance. Current block contract funding models also limit the extent to which CHS can provide the flexible services required if hospital admission is to be avoided. We also found some tension around the implementation of additional hospital avoidance services (eg, ‘virtual wards’) which did not necessarily integrate with or complement core CHS services.

**Conclusions:**

Our focus on the commissioning of CHS has highlighted the fact that the new collaborative approach to service design and delivery embodied by the creation of Integrated Care Boards has led to some confusion around decision-making. In addition, the lack of appropriate data and the funding and contractual model used to procure CHS impacts their ability to contribute to the policy agenda of treating more people in the community. These factors should be addressed if CHS are to fulfil ambitions of preventing hospital admissions.

**STRENGTHS AND LIMITATIONS OF THIS STUDY**

- A particular strength of this study was the voice it afforded participants, via qualitative interviews, in sharing their experiences of working in and/or for Community Health Services (CHS), which due to the paucity of research into CHS, have rarely been explored.
- The study was conducted during the implementation of Integrated Care Systems (ICSs) in England and the introduction of Integrated Care Boards and was therefore an opportune time to capture these changes from the perspective of CHS and to examine how this has impacted on their role in hospital avoidance.
- Recruitment of participants was challenging in some sites, and more providers of CHS than commissioners of CHS were interviewed. However, this reflects our findings regarding the maturity of ICSs and the current state of the commissioning function.

## Introduction

 In the context of an ageing population with increasingly complex needs, keeping patients out of expensive acute hospitals and providing more care in the community has been a policy aspiration in the UK and elsewhere for many years.^1^,^3^ However, despite this consistent policy focus,^4^,^6^ over at least the past 10 years, the National Health Service (NHS) in England has seen a relentless increase in secondary care activity, with primary and community care receiving a decreasing share of funding.^7^ The most recent set of NHS reforms is based on the idea that shifting away from competitive modes of healthcare organisation towards a more collaborative model will support the desired shift of more care into the community.^8^ The Health and Care Act 2022 (HCA22) created 42 Integrated Care Systems (ICSs) in England, bringing together the full range of service providers to collaboratively plan and manage services for geographical populations. It is argued that this more collaborative approach will facilitate more care being provided in the community, supporting a more proactive approach to provide more integrated and coordinated care at home.^8 9^ Most recently, the government has announced three major health ‘missions’: to shift care from hospitals into the community; to shift the focus of care from treatment to prevention; and to shift the NHS towards a digital approach.

Community-based services, including nursing care, physiotherapy and occupational therapy, for example, are crucial to this vision. In the UK, Community Health Services (CHS) are all health services provided to patients outside hospital apart from primary care (https://www.england.nhs.uk/community-health-services/). According to NHS Providers,^10^ CHS in England provide over 100 million patient contacts per year, account for an NHS budget of around £10 billion and constitute one-fifth of the total NHS workforce. Despite this significant volume of activity, and despite their importance to the achievement of policy aims, CHS are rarely the focus of policy^10^ or research.^11^ Moreover, data related to community service provision in England are underdeveloped.^12^ This lack of data makes it difficult to evidence the impact of community services on activity elsewhere in the system. What evidence there is suggests that increasing community-based activity does not reliably reduce activity elsewhere,^13^ but the reasons underlying this, and the extent to which these can be ameliorated, remain unclear. CHS are thus central to the achievement of policy aims but poorly understood in terms of their scope and impact.

In this paper, we seek to address this deficiency. Our study explored how CHS in England are being planned and managed to achieve the goal of optimising care outside hospital, with a particular focus on the factors which are supporting or impeding this work, in the context of a recent significant reorganisation of the NHS.

Our findings are relevant to ongoing policy and health system design in the UK and more widely, providing evidence about the factors that support or inhibit CHS providers in meeting the policy desire to provide care outside hospitals.

What follows is divided into five sections. An initial overview of what is meant by commissioning is followed by a short description of the reorganised NHS in England. We then present our methods, followed by the empirical findings structured around the elements of commissioning identified from the literature. Our discussion considers the implications of these findings for the planning and provision of services in the community to avoid unnecessary hospital admission.

### Commissioning

Commissioning refers to the process by which service provision is matched to population needs in a system in which planning and provision are separated.^14^ It can be conceptualised as a cyclical process of strategic planning for services including a systematic approach to needs assessment, service planning, contracting, monitoring and review.^15^ This has been the dominant mode by which services have been planned and procured in the NHS since the split between purchasing and provision was introduced in the early 1990s.^16^ While commissioning includes important transactional elements, such as letting and managing contracts, research has shown that commissioning also includes important elements of relationship management.^17 18^ This is particularly true in the context of systems seeking to facilitate collaboration and integration. A study of commissioning in the early 2000s^19^ generated a modified description of the important elements of commissioning for healthcare services:

- *Objective setting and decision making*, including: appropriate balance between national/regional and local objectives; mechanisms for setting those local objectives; clarity over the scope of decision-making powers vested in the commissioning authority; and governance structures by which they can be held to account for those decisions
- *Management of partnerships* across their geographical footprint, with recognition by partners of their legitimacy to do this
- *Supporting patient choice,* with this seen as the mechanism by which the public can influence the care that they receive
- *Information collection and analysis,* including: population health needs; local service maps; provider activity and quality data; patient satisfaction data; and intelligence about potential future factors likely to affect demand. Commissioners also need the analytic capability to understand trends and make sense of what the data is showing.
- *Service design and resource allocation*. Within this category, the authors highlight the need for commissioners to work closely with providers in service design decisions, and also the potential for some more specialised services to be designed and delivered over larger footprints by consortia of commissioners.
- *Procurement and contracting*, including: service specifications; contracting procedures (including competitive processes where relevant); contract monitoring; quality improvement; and performance management.

In the rest of this paper, we use these elements to interrogate the commissioning of CHS to support the avoidance of unnecessary hospital admission in the NHS in England. We explore the factors associated with the operation of the new system which are supporting or hindering this endeavour and draw conclusions relevant to other systems seeking to support a move to more out-of-hospital care.

### The integration agenda in England

The HCA22 built on a series of policy initiatives designed to promote integration between health sectors and between health and social care.^20^,^23^ It was argued that these initiatives had shown the potential for better integrated services that could reduce acute hospital activity, but that the previous system architecture had erected barriers between sectors and providers.^15^ The 2012 Health and Social Care Act focused on competition as the driver of quality improvement and sought to harness the knowledge of local clinicians to commission services for their local populations via 212 Clinical Commissioning Groups (CCGs).^24^ These organisations were given statutory responsibility for commissioning services from competing providers.

The new HCA22 reduced the requirement for competition and established 42 Integrated Care Systems (ICSs) across England which covered much larger populations than the CCGs that they replaced. ICSs were given four over-arching goals: to improve healthcare and population health outcomes; reduce inequalities in access, experience and outcomes; increase productivity and value for money; and help the NHS support social and economic development. ICSs consist of two main components: an Integrated Care Board (ICB), which carries the statutory responsibility for commissioning services and which brings together representatives of local providers; and an Integrated Care Partnership (ICP) which brings together NHS partners with representatives from the wider health and care system, including local authorities, the voluntary sector and wider agencies such as those housing and employment services. It is intended that collaboration between providers will facilitate the flexibility required to support the delivery of services which patients experience as joined up and seamless.^8^

In addition, ICSs were required to identify lower levels of organisation within their geographies, known as Places, which roughly correspond to the footprints of local authorities. Guidance suggested that most day-to-day decision-making about routine service provision and commissioning would be delegated to this level, with a particular focus on the facilitation of collaboration between primary, community, mental health and other services to keep people out of hospital.^9^ However, early evaluation has shown that this delegation has not yet occurred, with decision-making remaining centralised at the level of large scale ICBs, covering populations of over a million people.^25^ Importantly, although the HCA22 downgraded the importance of competition between providers, it left intact the notion of commissioning,^26^ by which the ICB contracts with a range of providers to provide services for their population. This means that, within an overall requirement to collaborate and integrate across sector boundaries, the processes and functions of commissioning (assessing needs, designing services to meet those needs, letting contracts to providers and monitoring performance) are still required. Moreover, ICBs are currently subject to planning for, and commissioning of services under difficult financial conditions, balancing constrained funding with ever-increasing pressure and demand for their services. Thus, ICBs face significant economic challenges in commissioning services for their populations within these parameters. Difficult decision-making in the pursuit of balancing budgets may impact not only the commissioning of local services, but also potentially undermine the basis for making funding decisions for commissioning based on need, and relationships with partners and service providers across ICSs that are central to the integration agenda.

CHS in England are provided by a mix of 'for profit' and 'not for profit' organisations, including: standalone community NHS trusts; combined acute/community NHS trusts (or NHS foundation trusts in either case); for profit companies and not-for-profit organisations such as Community Interest Companies.^27^ There is currently no good evidence about the advantages and disadvantages of these different ownership models.^28^

## Methods

### Sampling and recruitment

The aim of the study was to explore the commissioning of CHS and its effects on CHS organisation. Specifically, we set out to examine how decisions were made about matching supply of resources to (patient) demand, in order to help avoid hospital admissions, including funding allocation and associated decisions.

We undertook a series of case studies of CHS commissioning across four diverse geographical areas of England as part of a larger mixed methods study considering the role of CHS in avoiding unnecessary hospital admission.^27^ Case study sites were chosen on the basis of current supply and demand figures taken from analysis of the NHSE Community Services Data Set conducted by the quantitative arm of the study. We aimed for heterogeneity in both type of health economy (urban/rural), CHS organisational and ownership model, and the extent of matching of resources with demand: three sites with a mix of supply and demand which was relatively less well matched, equally matched or better matched (A, B and C in table 1). Site D acted as a triangulation site, allowing the testing of our findings in a different setting.

**Table 1:** Case study site characteristics

Site ID	Ownership model	Region	Area health economy/demographics	Current matching of supply (CHS staff) to (patient) demand
A	Provider partnership —including a mix of NHS and non NHS providers	South East	Mixed urban-rural	Average matched (ie, relatively average supply for population size compared with England as a whole)
B	Integrated with acute NHS Trust	North West	Urban	Better matched (ie, relatively high supply for population) size compared with the average across England)
C	Standalone Community Health Services Trust	South Central	Rural	Less well matched (ie, relatively low supply for population size compared with the average across England)
D	Integrated with acute NHS Trust	North	Mixed urban-rural	Better matched (ie, relatively high supply for population size compared with the average across England)

CHS, Community Health Services; NHS, National Health Service.

The study consisted of a series of interviews with individuals responsible for commissioning and providing CHS in each site. Participants were recruited via introduction following an initial scoping interview with the CHS gatekeeper who was usually a senior manager. This also enabled us to understand the local commissioning/provider landscape and organisation of the service and how and who to contact to request as participants. Prospective participants were then emailed and subsequent participants were recruited via snowballing from these contacts. These included a range of staff from both the provider and the associated commissioning organisation with knowledge of commissioning and contracting arrangements such as associate directors, senior commissioning managers, strategic planning directors, business analysts and community response leads. Final participant numbers were as detailed in table 2.

**Table 2:** Participant characteristics

Site	Providers	Commissioners	Total
A	9	1	10
B	7	10	17
C	7	3	10
D	3	0	3
Total	26	14	40

### Data collection

Semi-structured interviews were conducted by MG and DB (researchers with extensive experience of conducting, designing and analysing qualitative research projects), remotely via the Microsoft Teams platform with recorded, informed verbal consent from the participant and lasting an average of 50 minutes. An interview topic guide was used to structure the interview (see online supplemental material 1), which was derived from the research questions and previous research in the area. The topic guide was explicitly focused on the commissioning of services and not on the role of CHS more widely. Topics covered in the guide explored a range of questions regarding the commissioning and organisation of services and how decisions are made about matching supply of resources to (patient) demand to avoid hospital admissions, including funding allocation and associated decisions. The guide was supplemented with further topics as the interviews progressed and which were considered salient to investigate further. Alongside interviews, a case study description was created for each provider-commissioner dyad, which brought together details such as history and local geography, population details, organisation structure and strategy reports, for example. Field notes were also captured following interviews and discussed during the analysis process. NHS ethical approval was granted for the study (IRAS reference 321 707) along with approval from the University of Manchester Research Ethics Committee (reference 2022-15310-25431).

### Patient and public involvement

A front-line advisory group consisting of patient, public and CHS stakeholders was convened as a part of the wider mixed-methods study. Patients and the public were not involved in the planning or design of the study, but interim findings and reflections on consequences not observed by providers and commissioners were discussed with the group at quarterly meetings. No changes were made to the findings following these meetings, but the discussion was beneficial in confirming reflections and themes identified during data analysis, bringing additional rigour to the process. In addition, our patient and public involvement members will develop an accompanying output directed at providers and commissioners, reflecting their responses to the case study findings.

### Analysis

Reflexive thematic analysis^29^ was used along with the framework method to structure, organise and analyse the interview data^30^ and to enable cross-case comparison of the factors affecting the approaches taken to resource allocation and service planning, and the perceptions of respondents as to what works well and what could be improved. Analysis was conducted by DB and MG and involved discussion with the wider research team to ensure a reflexive approach to theme generation and interpretation of the data. Interview transcripts were uploaded to NViVO 14 qualitative data analysis software to aid coding of the data.

Each transcript was analysed using a coding framework which was developed both deductively from the topic guide and knowledge of existing literature as described below, and inductively, with additional codes being introduced from the data as the interviews progressed. Codes were then grouped into categories to explain concepts (common themes and patterns of shared meaning), occurring within and across the interviews and across the case study sites.

## Results

In this section, we set out the evidence from our case study sites. We have structured the findings around five overall themes derived from the commissioning literature: the overall organisation of commissioning, including who is responsible for decision-making; approaches to assessing needs; service design and development; contracting for services and funding; and performance management and support.

### Commissioning functions: who is responsible for what?

Inevitably, in the wake of a large-scale reorganisation, there was a considerable degree of confusion around how commissioning was being carried out. We spoke to both commissioners and providers who told us that organisational restructuring had led to a loss of skilled commissioning staff and that there was a lack of clarity about responsibilities:

…*I think in the setting up of the ICB, there hasn’t necessarily been a focus on commissioning, and I think we’ve lost sight of it a bit, and I’m not sure that we’ve got the right skills. I think commissioning is a very highly skilled thing to do, and I’m not sure that we’ve necessarily got a high level of skill within (site name), in terms of what mature commissioning really looks like*. (N0021re – Provider)

*I think there’s lots of conversations about funding and how we commission and all of that, but I’m probably not close enough to the root of that to actually understand what the future of commissioning would look like. I know there’s commissioners out there, but I don’t know what they commission, they don’t commission my services*. *(N*0017gd – Provider)

In part, this confusion arose with the fact that ICBs were given considerable latitude as to how they organise themselves to carry out their statutory functions.^9 31^ In addition, there was also some confusion about the concept of commissioning as a whole, with a perception from some that working more closely together meant that commissioning was no longer required:

*In terms of commissioning in general it kind of became a bit of a dirty word about 2 years ago, it was more…so we’re not going to have an old-fashioned commissioning and then contract and relationship, it’s going to be doing things in partnership*. (N0035ez – Commissioner)

However, others acknowledged that commissioning was still required:

*I think there’s been this view that we’re all in it together, and therefore we’re all responsible for commissioning services, whereas actually, while we would want to input our views, and we would want to be able to give our insights and knowledge—because we’ve got a huge amount of knowledge about our population—there’s no getting away from the fact that commissioning is still the responsibility of the ICB. So they can’t, kind of, divest themselves of that by saying, well, we’re all in it together, because we are, but we’re not. You know, they are still responsible for commissioning the services*. (N0021re – Provider)

As responsibility for commissioning formally shifted to ICBs (which cover a large geographical footprint), some providers noted that decision-making had become more remote, with CHS voices less likely to be heard:

*Even further, yes, absolutely, now that we’ve got a community collaborative governance system in place. But equally, you’d expect people that are attending those meetings to know what’s happening on the ground and to feed that up. Which they do, but from a service perspective, it just feels so much more removed by having that layer.* (N0041 – Provider)

Official guidance^9^ suggests that this will be addressed via the delegation of commissioning responsibilities back down to lower geographical levels, but in our case study sites at the time of data collection, there was confusion as to where these responsibilities currently sit. Commissioners remain employed at local levels, but their remit and decision-making powers are unclear:

*So even just now, 4 years after we landed, we are still looking at the governance role, trying to say, okay, if we're doing this, here’s our paper, we take it to the (name) exec but who ratifies it, who agrees it?* (N006jc *–* Commissioner)

### Assessing population needs

Against this background, we explored how the specific functions of commissioning were being carried out for CHS.

In assessing the extent of population need for services, the use of population health data was perceived as being vital to allow the identification of demand, one commissioning interviewee describing it as a ‘data driven approach to commissioning*’* (N006jc).

Population health and CHS data were being used for multiple purposes in planning and organising current and future services, including: identifying need; asset mapping in neighbourhoods; risk stratification to determine priorities; management capacity; and matching staff resources to service demand for current services. All of our case study sites were auditing and prioritising CHS offerings across their Places and neighbourhoods according to needs assessment and in attempts to address service variation:

*In a business model you wouldn’t provide a service to everybody just because they’re over a certain age whether they’re going to access it or not, you’d actually look at your demographic. So, I’m saying we’ve got to have the Population Health data is key for us to be able to commission a service that everybody can access who needs it. But not everybody needs it. So, we need to really understand who in our population needs it and commission for that cohort*. (N005lu – Commissioner)

However, much of this population health data is not in a readily usable form, with sites describing the complexities of drawing on multiple sources of health and social care data (public health, primary care, CHS, social care, secondary care, ambulance service), from separate organisations, systems, tools, platforms and dashboards, to provide accurate, fine-grained identification of need. Some sites were further along this journey than others, nonetheless we identified frustrations about the lack of usable data.

Both commissioners and providers expressed a desire to more appropriately match the supply of services to some measure of need, but how ‘need’ was defined varied. Commissioners tended to speak about population need; while providers were mindful of this, they also defined ‘need’ in terms of demand on their services as expressed by actual use. However, CHS data are difficult to collect and often fail to capture the nuances of CHS activity, making it difficult for providers to evidence how stretched they were:

*The time we’re spending with patients and the complexity and the length of time they’re on our caseloads have increased. So it’s really difficult to demonstrate sometimes that that increasing demand….So it’s the incidental data, the soft data, sometimes that’s really hard. Cause I think still there’s a bit of a concentration on…although it’s not meant to be, on raw data and data that’s, like, how many contacts have you had, how many referrals, how many discharges…and we were told that it was moving away from that a bit, but it feels like it hasn’t quite got there yet.* (N0028ru – Provider)

Participants mentioned that, aside from needing good data, good analytical skills and joined up access to it, this must be translated into action with the corresponding financial and staff resources allocated to services:

*We can all agree based on the needs of population and I think we’re really strong on that side of it, on understanding what the population for each of the (n) localities, but also across (site name), in what we need. I think but when you start to talk about, okay, how are we going to do it, who’s going to do it and who’s going to pay for what, it falls down a little bit.* (N0035 *– Co*mmissioner)

### Designing and developing services

It has always been the case that the design of services to meet identified population health needs required collaboration between commissioners and providers, not least because, in general, it is providers who have the fine-grained knowledge of services that is required.^32^ Many respondents were enthusiastic about the opportunities that the new collaborative approach afforded:

*There was a lot more of a, sort of, bureaucratic cycle, for want of a better description, that we would go through, whereas, now, we’re really engaging with the providers and trying to encourage them to be innovative, come up with solutions, you need it, we’ll follow through, we’ll try and work out how that could be commissioned and whether you’re the right provider for it. As opposed to us leading it, so it’s much more of a partnership approach*. (N0027ml – Commissioner)

At the same time, as the reorganisation into ICSs disrupted local teams of commissioners, providers reported that they were being asked to do more of the service design tasks:

*What [commissioning resources] aren’t there now is being pushed out to providers, so, it’s…the ask on us has become much more.* (N0029df – Provider)

Thus, in some areas, CHS providers had stepped in to fill the vacuum by taking on some functions, such as communicating service changes to primary care colleagues. In addition, the disruption of local commissioning teams had undermined longstanding relationships and opportunities for providers to make business cases for extra funding:

*That direct clinician conversation with a Commissioner has gone. (N0041c*v – Provider)

This shift of service design responsibilities to providers produced complexities in relation to the appropriate footprint across which services should be planned. As discussed above, population health need and the associated need for CHS are driven by many factors, including deprivation, illness prevalence and the availability of social care and voluntary sector services. These often vary across small geographical areas, and commissioners acknowledged the need for fine-grained analysis of need over small populations often identified as ‘neighbourhoods’ or ‘places’. Most providers of CHS in the NHS, by contrast, tended to cover geographical footprints considerably larger than the geographical populations represented by previous commissioning organisations (ie, CCGs) and current places.

Thus, there was a potential for tension between two different drivers: a provider-driven desire for consistency across their service footprint to improve equity and efficiency, and a recognition that commissioning needed to focus on adapting services to meet local needs across smaller geographical areas. This tension may be compounded by current confusion as to how far responsibilities are to be delegated from the ICB to lower geographical levels.

Providers wanted to develop a ‘core offer’ which was the same across their entire footprint, with the potential for local variation at the margins:

*…so, we’re in the process of doing it, we haven’t landed it just yet, but we’re in the process of developing, kind of, a consistent integrated community core offer. So, that will be consistent across each of our (n) ICT footprints*. (N033qj – Provider)

*So, you have your core offer but then you're able to start based on the needs of the population, develop your service offer in that direction, and that we want our offer to be more flexible and agile in how it works*. (N004uu – Provider)

Optimising these local variations will require clarity about responsibility for decision-making for local populations, as it is possible that a large provider covering more than one ICS area will have a different view of what is needed than those responsible for particular local populations at Place or neighbourhood level. The optimum footprint across which CHS should be designed and planned is not known, nor is there any agreed consensus over what a ‘core offer’ of community services should be.^33^

In keeping with the lack of clarity over what services CHS should provide for which populations, we found that local determination of service design was overlaid with nationally mandated services specifically focused on trying to keep people out of hospital. These are set out in the national Priorities and Operational Planning Guidance which ICBs must follow.^34^ These included so-called ‘Virtual Wards’ and Urgent Care Response teams, focusing on providing additional intensive home-based services to keep people at home. ICBs were required to implement these alongside their local priorities for CHS. While interviewees understood the rationale for such initiatives, some also described them as taking time and attention away from efforts to optimise their standard service offer. They also required staff and resources to be diverted to teams which were not necessarily fully integrated with the standard service:

*There’s obviously urgent community response and there was some national funding that came down from government around urgent community response, but that really equated to an additional nurse, an additional therapist and some admins. That’s all that money equated to. From a service manager point of view, we were then asked to find resource within our current staffing establishment, really, to be able to deliver a service*. (N0039fx - Provider)

There was also a risk that these separately established services could lead to fragmentation:

*Because what we have at the moment is very fragmented. There’s a lot of funding being given to virtual frailty wards, which is work that bread and butter community nurses have done for years anyway. And actually, if you just enhanced those teams, you would actually give those nurses more time to do what they need to do, because you wouldn’t be doubling up. At the moment you could have a UCRT nurse going in, a virtual frailty nurse and a district nurse going into one person’s home. Why would you do that? But I’ve been told very clearly, they have to stay as three separate teams, and it makes no sense to me*. (N0029df – Provider)

Hence, CHS providers tend to see all of their services as relevant to the need to keep people at home, whereas national policy has tended to focus on additional initiatives. Moreover, admission avoidance is potentially difficult to evidence. While separately established services such as Virtual Wards or Clinical Navigators (staff who may be positioned at the (real or virtual) hospital ‘front door’ to identify and divert patients from the emergency department to the appropriate community service), can demonstrate their value in preventing admissions, it is significantly harder for routine home-based services to do the same, as it is often only in retrospect and at local level that a particular care episode can be seen to have enabled a patient to remain at home. This means that local conversations about where to invest or deploy resources in order to bring about the desired shift away from admissions to hospital are hampered by lack of evidence of the impact of routine community services. This in turn ensures that standalone services are more likely to attract investment than more routine standard services.

### Contracting and funding

In part because of the difficulties associated with the available activity and outcomes data, CHS in England are purchased through ‘block contracts’ which provide a fixed budget for services which does not increase if activity increased above an agreed level. This representative from a CHS provider part of an acute NHS Trust explained that they were required to account to the Trust finance manager for their activity beyond their contract:

*In some parts of the city, we over-perform, by which I mean we provide more activity that we’re commissioned for, ‘cause we think it’s the right thing to do and that’s painful because it means that, when we account to [acute Trust] for our budget, we have to explain why it is that we are over-performing in terms of the commissioned levels of activity, and that’s differential across three localities*. (N002mk – Provider)

Due to the form of contract used, providers were not given additional resources to pay for increased workload so that they had to absorb this cost. Although the national admission avoidance schemes such as Virtual Wards received some additional funding, this was felt not to represent the true costs of providing the services, and obtaining additional investment for core services to support care outside of hospitals, was hampered by the block contract and by the identified difficulties in evidencing impact.

### Monitoring and support

Alongside the lack of clarity as to exactly where commissioning responsibilities sit within the new system, we found a lack of clarity around performance monitoring. Some sites told us that performance and quality monitoring had continued as before, while others said that since the reorganisation monitoring had fallen away:

*There’s certain things that aren’t getting done. I’ve certainly never had a performance meeting, apart from with NHS England for vaccinations, for community health. I mean there’s some high-level stuff that goes on in the system. But I would normally be used to working alongside and looking at where our hotspots are, trying to pick them apart, looking collectively at how we could improve on those. We don’t have any of that oversight at the moment. So I’d say that there are definitely jobs that aren’t getting done*. (N0030jx – Provider)

Some sites described a move from CHS providers being directed to deliver specific services and closely monitored (via activity-focussed, measurable performance targets such as Key Performance Indicators and reporting), to a focus on the codesign of services and ‘light touch’ assurance:

*Even for the ICB they're in unchartered waters. So even the commissioners themselves, they don’t function like they previously would have, to say, for example, I have a pot of money, I want to deliver X type of service, I will commission that. That kind of role, it’s not quite there anymore. We’re not commissioning it as was, we’re just deploying almost*. (N0014bx – Commissioner)

It was thus unclear how problems in either service quality or volume of activity would be detected and addressed, and by whom.

## Discussion

Our study explored in depth the new arrangements for commissioning and provision of CHS and community nursing services following a significant reorganisation of the NHS, with a focus on understanding how the policy priority of reducing avoidable hospital admissions was being considered and planned for. While it could be argued that some of our findings arise out of the inevitable disruption and loss of performance associated with reorganisations,^35^ there are some more general lessons that can be drawn. First, notwithstanding the desire for greater collaboration, our study suggests that however services are planned and managed, clarity is required over who is responsible for what. This chimes with the literature on integrated care, which highlights the fact that while outcomes such as keeping people out of hospital require action across sectors, it is important that alongside clear shared goals, there is a good understanding of the roles and responsibilities of individual organisations within the collaborative setting.^36 37^

Relatedly, we have highlighted confusion over the meaning of commissioning in the reorganised system. This has wider implications beyond the NHS in England. There is a limited number of ways that services can be planned and paid for. The NHS in England currently relies on capitated budgets covering administrative areas, within which services are provided via contracts between a commissioning authority and a population of providers. An alternative approach would be to return to a more directly managed system as existed prior to 1991, in which local planning authorities were responsible for the provision of services to their local population via directly managed hospitals and community-based services.^38^ That this was not done under the Health and Care Act 2022 implies that those responsible continued to see some value in a contract-based system. However, the reorganisation left those affected in some doubt about how far contracts and contract management were important, as well as confusion over who was responsible for service design. This suggests that whatever mode of planning is used, if policy priorities are to be realised, clarity is required over how decisions are to be made, how services are to be monitored and how funds are to be allocated between sectors.

The lack of good quality data for CHS services delivery has been long known but slow to be remedied.^13^ Any improvement in the ability of CHS to deliver appropriate and cost-effective out of hospital care will require access to better data, and our study suggests that the pooling of expertise and data between different parts of the system would be useful. Service design was also complex in our study, with tension between delivering national requirements and prioritising local service design to meet local needs. In particular, we found that, in pursuit of the overall policy goal of keeping people out of hospital, there is no consensus around the right balance between investing in standard community-based services and funding additional ‘add on’ services such as Virtual Wards, and this is an area in which further research would be useful, although dependent on the availability of appropriate data.

In addition, the desire of providers to deliver largely uniform services across their large footprint needs to be reconciled with the commissioning imperative to deliver services that meet the unique needs of local populations. This challenge is not unique to the UK, and further research is required to clarify both the optimum size of population for whom CHS should be planned, and the menu of services which are most likely to enable an ageing population to remain independent in their own homes as long as possible.

The tension between national ‘top down’ requirements and the need for local service design requires consideration, as illustrated in this study. The NHS is a highly centralised system, and past attempts to increase local autonomy have rarely succeeded.^39^ In addition, the intention is that ICBs should delegate service design and commissioning to local geographies such as Places and neighbourhoods^9^ and that local areas should have the autonomy to work flexibly together to deliver integrated services across boundaries; our study suggests that if this is to become a reality, clearer guidance about how delegation of responsibilities within ICBs should occur is needed, alongside willingness from national authorities to trust local areas.

Finally, how best to fund CHS so that they can respond flexibly to fluctuating demand remains an important question. This is particularly important when, as is currently the case, resources are significantly constrained. In the Netherlands, independent nursing teams funded to plan and deliver services, linking closely with community groups and the voluntary sector, have delivered remarkable outcomes, including reduction in hospital admissions,^40^ but attempts to replicate this in the UK have been less successful, in part due to cultural and regulatory differences.^41^ While intuitively it might seem feasible to move money from hospital care to community services where such services are successfully keeping people out of hospital, in practice, this has proved very difficult to achieve,^42^ and this is borne out by our study, where even those providers providing both hospital and community services seemed to find it difficult to move money between sectors. It may be that if funding overall were to be increased, these issues would become easier to manage. However, it seems unlikely that funding will improve in the short or medium term.

### Strengths and limitations

A particular strength of this study is that it presented an opportunity to explore the work of CHS, which are an under-researched, yet critical component of the healthcare system. Little work has been conducted in this area, and as such, this study therefore serves to inform policymakers and stakeholders alike of the experiences of CHS in their endeavour to integrate services, to shift more care out of hospital and into the community and prevent hospital admissions. Additionally, the study was conducted during the implementation of ICSs in England and the introduction of ICBs, and it was therefore an opportune time to capture these changes from the perspective of CHS. In terms of limitations, we did not reach the desired cohort of commissioning and provider manager dyads. Recruitment of participants was difficult in some sites, and more providers than commissioners of CHS were interviewed. We speculate that this was because of the state of flux that the system was going through with the subsequent organisational redesign of systems from CCGs to ICBs, and it may be related to our finding that providers were often unable to identify who their local commissioners were. This meant that commissioners were hard to locate, but conversely providers were keen to tell their story. This, however, reflects our findings regarding the maturity of ICSs and the current state of the commissioning function. Given the small-scale nature of our study, findings cannot be generalised to all providers and commissioners of CHS.

### Conclusion

Given the importance of CHS to the policy agenda of increasing proactive care outside of hospitals,^8 9 31^ our findings shed light on factors that support or inhibit this aim. Despite being 2 years old, the move to ICSs is still bedding in, and this is impacting the ability of CHS to fulfil their role in reducing avoidable hospital admissions and enabling people to remain at home. We heard many examples where this work is being conducted, whether through national initiatives such as Virtual Wards, or through the core CHS offering, but this was impacted by lack of good data, lack of clarity about roles and responsibility within systems and, most importantly, inflexible funding models which fail to support shifts in services. Improving the ability of CHS to provide proactive care in the community will require attention to each of these factors.

## Supplementary material

10.1136/bmjopen-2024-098159 online supplemental file 1

## Data Availability

Data are available upon reasonable request.
