# Supplementary material for: ‘We’re not there yet!’: a qualitative study exploring the commissioning of adult Community Health Services in England to support the avoidance of hospital admissions
Source: BMJ Open. 2025 May 31;15(5):e098159. doi: 10.1136/bmjopen-2024-098159 (PMC12142099; doi:10.1136/bmjopen-2024-098159)
Supplement: online supplemental file 1 [file bmjopen-15-5-s001.docx]

## Understanding the role of adult community health services in avoiding hospital admissions

## Topic guide

Interviews with both commissioners and providers explore the following questions:

(Note: use questions appropriate to participant)

- Please tell me a little bit about your role.
- To what extent and how do commissioners/providers define ‘need’ for community services?
- How do services get planned and then commissioned?
  - Core services – what are they?
- Has commissioning and how you work with commissioners/providers changed?
  - If so, how?
  - What is the decision making process?

- Are there any specific plans underway aimed at reducing hospital admissions?
- How are funding decisions made?
  - How does it get apportioned?
- What contractual mechanisms are used to deliver community services?
- What about resources - how are decisions made in terms of matching supply and demand?
  - How do commissioners allocate resources to match supply with demand.
  - How might the allocation of resources be improved to better match supply with demand?
- How do commissioners monitor levels of activity and quality of service delivery?

(or)

- How do providers monitor service performance?
- To what extent and how are these mechanisms and processes used to avoid admissions?
- What factors have determined the approaches used, including integration across health and social care boundaries?
- What outcomes do commissioners and providers value beyond admission avoidance?
- **Is there anything else helpful for us to know that we have not covered today?**
- **Is there anyone else we should speak to?**

**Thank you for your time today!**
