# Supplementary material for: ‘We’re not there yet!’: a qualitative study exploring the commissioning of adult Community Health Services in England to support the avoidance of hospital admissions
Source: BMJ Open. 2025 May 31;15(5):e098159. doi: 10.1136/bmjopen-2024-098159 (PMC12142099; doi:10.1136/bmjopen-2024-098159)
Supplement: Supplementary file 2 [file bmjopen-2024-098159.reviewer_comments.pdf]

PEER REVIEW HISTORY

BMJ Open publishes all reviews undertaken for accepted manuscripts. Reviewers are asked to complete a checklist review form and are provided with free text boxes to elaborate on their assessment. These free text comments are reproduced below.

ARTICLE DETAILS

Title (Provisional)

'We're not there yet!' – a qualitative study exploring the commissioning of adult Community Health Services in England to support the avoidance of hospital admissions

Authors

Bramwell, Donna; Goff, Mhorag.; Allen, Pauline; Meacock, Rachel; Checkland, Kath

---

VERSION 1 - REVIEW

**Reviewer**	**1**
**Name**	**Junghans, Cornelia**
**Affiliation**	**Imperial College London, Department of Primary Care and Public Health**
**Date**	**15-Feb-2025**
**COI**	**None**

This is an important and timely subject and a well written paper.

My only suggestion would be to clarify the constraints of funding to the ICS by NHSE and how this might help or constrain impact local commissioning.

There is a suggestion that it is the regulatory and cultural differences that mean that

The paper is focussed on CHS but perhaps it could be set into the wider context of the ICS, with an acknowledgement that some of the challenges go beyond commissioning within the CHS, particularly when it comes to reducing hospital admission, increasing fairness and improving outcomes. For example Adult Social Care paid for through local government has a major impact on hospital admissions and discharge. The complexity in commissioning services in the locality is increased by the fact that there is still silo'd commissioning between CVS, local authority, GPs, CHS for outcomes everyone is jointly responsible?

Are there any recommendations the authors can make on how to remove some of these barriers based on their work?

Minor comment: there seems to be a type in line 7 'by' and 'through' should it be either or?

**Reviewer**	**2**
**Name**	**Girling, Melissa**
**Affiliation**	**Northumbria University**
**Date**	**07-Apr-2025**
**COI**	**None**

This is an interesting paper and one which covers an important topic. Overall the paper is presented well and makes valuable points that will contribute to the field. My one question relates to how (if any) data relating to 'other' themes outside of the commissioning literature were dealt with? Were other 'less common' themes identified, and if so, could these be incorporated into the analysis? The exclusion of such themes could potentially add new knowledge outside of the scope of the current literature particularly as the authors note that this is an under-researched field of enquiry. A minor revision might be to include for example, acknowledgment of such data (if appropriate) and/or justification as to why this is not included? This could potentially move the field further on.

For PPI involvement - the authors note that interim findings 'were discussed with the group at quarterly meetings'. Could the authors elaborate on whether, for example, any changes were made to the findings as a result of these discussions and/or how this enhanced the analysis?

VERSION 1 - AUTHOR RESPONSE

- ***Reviewer: 1 - Dr. Cornelia Junghans, Imperial College London:***

1) **Comment:** My only suggestion would be to clarify the constraints of funding to the ICS by NHSE and how this might help or constrain impact local commissioning.

   Response: A sentence clarifying the situation regarding funding has been added to Page 5 and has then also been referred back to in the Discussion on Page 13.

2) **Comment:** There is a suggestion that it is the regulatory and cultural differences that mean that the paper is focussed on CHS but perhaps it could be set into the wider context of the ICS, with an acknowledgement that some of the challenges go beyond commissioning within the CHS, particularly when it comes to reducing hospital admission, increasing fairness and improving outcomes. For example Adult Social Care paid for through local government has a major impact on hospital admissions and discharge. The complexity in commissioning services in the locality is increased by the fact that there is still silo'd commissioning between CVS, local authority, GPs, CHS for outcomes everyone is jointly responsible? Are there any recommendations the authors can make on how to remove some of these barriers based on their work?

   Response: Thank you for drawing our attention to this. A theme throughout our data was the complexities involved with cross sector working,

particularly with regard to funding and the need for good quality data for commissioning. Our recommendations for action are detailed on Page 14 and we have also added a sentence in the Discussion on Page 14 acknowledging the need for clarity in both shared and individual organisational goals, roles and responsiblities towards collaborative action in avoiding hospital admissions.

3) **Comment:** Minor comment: there seems to be a type in line 7 'by' and 'through' should it be either or?

Response: The word 'through' has been removed. Thank you for noticing this.

- ***Reviewer: 2 - Dr. Melissa Girling, Northumbria University - Comments to the Author:***

1) **Comment:** This is an interesting paper and one which covers an important topic. Overall the paper is presented well and makes valuable points that will contribute to the field. My one question relates to how (if any) data relating to 'other' themes outside of the commissioning literature were dealt with? Were other 'less common' themes identified, and if so, could these be incorporated into the analysis? The exclusion of such themes could potentially add new knowledge outside of the scope of the current literature particularly as the authors note that this is an under-researched field of enquiry. A minor revision might be to include for example, acknowledgment of such data (if appropriate) and/or justification as to why this is not included? This could potentially move the field further on.

Response: Thank you for this very insightful suggestion. The study was part of a wider mixed methods study consisting of 5 workpackages looking at CHS and avoiding hospital admissions. The focus of the qualitative workpackage was explicitly on commissioning. We acknowledge that there are clearly wider questions on the organisation of, and the role of CHS, but as this was not the focus of the study, our interview topic guides focused explicitly on questions about commissioning and thus themes identified in the analysis, reflect this. We have noted this in the Data Analysis section on Page 7.

2) **Comment:** For PPI involvement - the authors note that interim findings 'were discussed with the group at quarterly meetings'. Could the authors elaborate on whether, for example, any changes were made to the findings as a result of these discussions and/or how this enhanced the analysis?

Response: Interim findings were discussed with the wider project team which included patient and public and CHS stakeholders. No changes were made to the findings but it was beneficial to talk to those with experience of CHS to affirm that themes and reflections identified during the analysis process made 'analytical sense' and to draw on their expertise in confirming and understanding the salience of those themes. A sentence to this effect has been added to the PPI section on Page 7.

VERSION 2 - REVIEW

**Reviewer**	**1**
**Name**	**Junghans, Cornelia**
**Affiliation**	**Imperial College London, Department of Primary Care and Public Health**
**Date**	**27-Apr-2025**
**COI**	

I'm happy with the revised manuscript which adequately takes into account suggestions made and concerns raised.
